# Correction: Green synthesis of nitrogen-doped multiporous carbons for oxygen reduction reaction using water-caltrop shells and eggshell waste

**DOI:** 10.1039/d1ra90116h

**Published:** 2021-05-21

**Authors:** Chun-Han Hsu, Zheng-Bang Pan, Hau-Ting Qu, Chuan-Ren Chen, Hong-Ping Lin, I-Wen Sun, Ching-Ying Huang, Chun-Han Li

**Affiliations:** General Education Center, National Tainan Junior College of Nursing Tainan 700 Taiwan; Department of Chemistry, National Cheng Kung University Tainan 70101 Taiwan hplin@mail.ncku.edu.tw iwsun@mail.ncku.edu.tw +886-6-276-2331 +886-6-275-7575 ext. 65342; Green Energy and Environment Research Laboratories, Industrial Technology Research Institute Tainan 71150 Taiwan

## Abstract

Correction for ‘Green synthesis of nitrogen-doped multiporous carbons for oxygen reduction reaction using water-caltrop shells and eggshell waste’ by Chun-Han Hsu *et al.*, *RSC Adv.*, 2021, **11**, 15738–15747. DOI: 10.1039/D1RA02100A.

The authors regret that there was an error in the funding information in the original article. The grant number was given as 108-D0111 in the original article, where it should have read 110-D0111.

The Royal Society of Chemistry apologises for these errors and any consequent inconvenience to authors and readers.

## Supplementary Material

